# Development of a nomogram based on serum cytokine-related riskscore in breast cancer

**DOI:** 10.3389/fonc.2023.1146463

**Published:** 2023-03-07

**Authors:** Ye Zhu, Yang He, Chong Chen, Jingyi Zhang, Xin Yang, Yuqing Lu, Yong-Zi Chen, Weipeng Zhao

**Affiliations:** ^1^ Department of Breast Oncology, Tianjin Medical University Cancer Institute and Hospital, National Clinical Research Center for Cancer, Tianjin, China; ^2^ Key Laboratory of Breast Cancer Prevention and Therapy, Tianjin’s Clinical Research Center for Cancer, Tianjin, China; ^3^ Key Laboratory of Cancer Prevention and Therapy, Tianjin’s Clinical Research Center for Cancer, Tianjin, China; ^4^ Department of Breast Cancer, Tianjin Cancer Hospital Airport Hospital, Tianjin, China; ^5^ Department of Clinical Laboratory, Tianjin Medical University Cancer Institute and Hospital, National Clinical Research Center for Cancer, Tianjin, China; ^6^ Tianjin's Clinical Research Center for Cancer, National Human Genetic Resources Sharing Service Platform, Tianjin, China; ^7^ Laboratory of Tumor Cell Biology, Tianjin Medical University Cancer Institute and Hospital, National Clinical Research Center for Cancer, Tianjin, China

**Keywords:** cytokine, riskscore, nomogram, breast cancer, prognosis

## Abstract

**Background:**

Cytokines are involved in many inflammatory diseases and thus play an important role in tumor immune regulation. In recent years, researchers have found that breast cancer is not only related to genetic and environmental factors, but also to the chronic inflammation and immunity. However, the correlation between serum cytokines and blood tests indicators remain unclear.

**Methods:**

A total of 84 serum samples and clinicopathological data of breast cancer patients from Tianjin Cancer Institute & Hospital, Tianjin Medical University, Tianjin, P. R. China were collected. The expression levels of the 12 cytokines were detected by immunofluorescence method. Blood tests results were obtained from medical records. By stepwise Cox regression analysis, a cytokine-related gene signature was generated. Univariate and multivariate Cox regression were used to analyze the influence on the prognosis of patients. A nomogram was constructed to illustrate the cytokine-related riskscore predicting 5-year OS, which was further evaluated and validated by C-index and ROC curve. The correlation between the expression of cytokines in serum and other blood indicators was studied by using Spearman’s test.

**Results:**

The riskscore was calculated as IL-4×0.99069 + TNF-α×0.03683. Patients were divided into high and low risk groups according to the median riskscore, with the high-risk group has a shorter survival time by log-rank test (training set, P=0.017; validation set, P=0.013). Combined with the clinical characteristics, the riskscore was found to be an independent factor for predicting the OS of breast cancer patients in both training cohort (HR=1.2, P<0.01) and validation cohort (HR=1.6, P=0.023). The 5-year C-index and AUC of the nomogram were 0.78 and 0.68, respectively. IL-4 was further found to be negatively correlated with ALB.

**Conclusion:**

In summary, we have developed a nomogram based on two cytokines including IL-4 and TNF-α to predict OS of breast cancer and investigated their correlation with blood test indicators.

## Introduction

According to data released by the International Agency for Research on Cancer (IARC) in 2020, breast cancer has surpassed lung cancer for the first time, becoming the world’s most common cancer, accounting for approximately 11.7 per cent of new cancer cases. Breast cancer is not only the most common cancer in women, but also the leading cause of cancer-related death, which seriously threatens women’s life and health. Breast cancer is a disease possessed different morphology, molecular characteristics, behaviors, and therapeutic responses (1). There are many traditional clinical pathological variables and molecular predictive markers to reflect the prognosis. The feasibility of serum tumor markers in the diagnosis, prognosis and clinical treatment of malignant tumors has been extensively studied nowadays.

Cytokines are small molecular glycoproteins that are synthesized and secreted by different cells (mainly monocytes and activated T lymphocytes) in response to stimulation. Cancer cells can also produce cytokines. When cytokines affect the growth and function of immunocompetent cells, they can activate or modulate the anti-tumor response and act to promote or inhibit tumor growth (2).The serum levels of IL-6, IL-8 and Il-10 may be used as potential markers to predict the metastasis and prognosis of breast cancer in a Chinese population study (3). But the effect of cytokine expression on the prognosis of breast cancer patients is still controversial. IL-6 is one of the most studied cytokines in breast cancer. A meta-analysis of 3224 breast cancer patients showed that IL-6 expression was not associated with DFS (4). Though Ahmad N and his colleagues demonstrated that high IL-6/IL-10 expression was associated with better disease-free survival (5). And another study found that circulating IL-6 is associated with poor survival in patients with metastatic breast cancer (6). A study on TNBC indicated that simultaneous inhibition of IL-6 and IL-8 expression significantly inhibit tumor growth *in vitro* and *in vivo*. Multivariate Cox analysis showed that IL-6 and IL-8 expression can predict the survival time of patients (7). Increased serum IL-8 expression in patients with advanced breast cancer and multivariate analysis showed that serum IL-8 was an independent significant factor affecting the post relapse survival (8). Furthermore, in a study of 207 patients with invasive breast cancer, high IL-17 status was found to be a significant prognostic factor for DFS (9).

The presence of inflammatory cytokines in the tumor environment affects a series of processes at different stages of tumor progression, including initiation, proliferation, promotion, transformation, angiogenesis, invasion, inhibition of apoptosis, immune surveillance, drug resistance and metastasis (10). There was also a correlation between blood tests indicators and prognosis in breast cancer patients. Liu’s team found that ALB, LDH and total bilirubin were significantly associated with 5-year OS. Patients with higher albumin levels, higher total bilirubin levels, and lower LDH levels had a lower risk of death (11).A meta-analysis revealed a significant effect of higher serum LDH on poorer overall and progression-free survival (12). Therefore, the correlation between cytokines and blood indicators is worth exploring. Ultimately, we examined 12 different cytokines in the serum level and explored their prognostic impact, then analyzed their correlation with routine blood tests and liver kidney function tests. We hope our findings could serve to shed some light on the prognosis of breast cancer patients.

## Materials and methods

### Samples and clinical data collection

A total of 215 patients with available serum sample were initially included in our study from 2003 to 2020 in Tianjin Cancer Institute & Hospital, Tianjin Medical University, Tianjin, P. R. China.

According to the following inclusion criteria (1): pathological diagnosis of breast cancer (2); complete clinical and pathological records (3); no treatment or exclusion criteria (1): any acute or chronic infection (2); combined with other types of primary malignant tumors; and (3) patients with bilateral primary breast cancer, 131 patients were excluded and 84 samples were included in the study. The Medical Ethics Committee of our institute approved this study (ethics number E2013134).

The results of liver and kidney function tests (ALB, GLO, A/G, ChE, ADA, LDH, GPDA, 5’NT, LAP, β2-MG), routine blood examinations (NEUT, EO, BASO) and coagulation factor D-dimer from 84 blood samples were obtained from medical records. OS was defined as the time from the date of diagnosis to death or censoring. (The follow-up ended on 30 April 2021).

### Measurement of cytokines

Venous blood samples were collected with standard test tubes, naturally solidified at room temperature or centrifuged at 2000-4000 rpm for 20 minutes, and about 0.5 mL of isolated serum was taken for examination. A Combined Cytokine Detection Kit (Ceger Biology) was adopted to simultaneously analyze the concentrations of 12 different cytokines in the serum of 84 samples following the manufacturer’s recommendations, in a flow cytometer. This kit is based on immunofluorescence technique. The mixture of capture microspheres contained 12 kinds of capture microspheres with different fluorescence intensities. The surface of capture microspheres was coated with 12 different cytokine-specific antibodies, the capture microspheres were specifically bound to the cytokines in the samples to be tested, and then combined with the PE-labeled fluorescent detection reagent. The capture microspheres, detection reagent, and detection antibody formed a double-frame sandwich complex, by analyzing the fluorescence intensity of the complex, the content of cytokines in the samples to be measured was obtained. The 12 cytokines included:IL⁃1β, IL-2, IL-4, IL-5, IL-6, IL-8, IL⁃10, IL-12p70, IL-17A, TNF-α, IFN-α and IFN⁃ γ.

### Construction and validation of cytokine-related signature

Using the “sample” function of R, the cases were divided into training set (n = 58) and validation set (n = 26) by 7:3 random sampling without return. In the training set, with serum expression levels of the 12 cytokines as independent variables and overall survival as the outcome, optimized by Cox regression analysis using the “step” function of R, screening for cytokines that are strongly associated with overall survival in breast cancer. The median riskscore was used as the cut-off value to divide breast cancer patients into high-risk group and low-risk group. Log-rank tests and Kaplan-Meier curves were produced using the “survival” and “survminer” packages. The riskscores of the validation cohort were computed with the unified formula established in the training cohort. In the aftermath, subgroup survival analyses were performed on BC patients with different clinical characteristics (age, tumor stage and molecular subtype) to further assess the predictive value of the riskscore.

### Construction of nomogram for breast cancer patients based on cytokine-related riskscore

In both training and validation sets, Cox regression analysis was performed with age, clinical stage, molecular subtype, and cytokine-related riskscore as independent variables, and overall survival as the outcome to construct a prognostic prediction model for breast cancer. Hazard ratio (HR), 95% confidence interval (CI), and p-values were separately determined. The “rms” package was applied to create a nomogram containing cytokine-related riskscore to predict the OS of BC. The C-index and score of the nomogram were calculated using the “survcomp” and “nomogramFormula” packages, respectively. ROC curves were plotted using the “timeRoc” package to further evaluate the predictive performance of the nomogram, riskscore and other clinical indicators.

### Statistical analysis

All analyses were performed using IBM SPSS software 25.0 and R 4.2.0. The Chi-square tests were used to compare the clinicopathological characteristics between the training and validation sets. Youden index was used to determine the optimal cut-off point to divided IL-4 and TNF-α into low and high expression group. Univariate and multivariate Cox proportional hazards models were used to analyze prognostic risk factors. Differences in OS in subgroups were calculated using Kaplan-Meier plots and log-rank test by SPSS. We made a thermogram of the correlation between IL-4, TNF-α and blood test indicators by R (4.2.0).Then we used the Kaplan-Meier plotter database (13) (http://kmplot.com/analysis/ ) to calculate OS according to gene expression levels by using specific probes in different datasets. According to IL-4 expression level (Affymetrix ID, 207539_s_at; dataset, GSE20711), a total of 88 breast cancer patients were included. And TNFA (Affymetrix ID, 207113_s_at; dataset, GSE1456) involved 159 patients. All splitting patients by auto select best cutoff. A P-value < 0.05 was considered statistically significant. Because the data are randomly lost, the missing values (< 5%) of covariates are replaced by average values.

## Results

### The clinicopathological data

Based on the inclusion and exclusion criteria, a total of 84 blood samples were included in the study. They were divided into training set (n = 58) and validation set (n = 26). 28 (48.3%) patients were older than 53 years old in the training set (median age of patients in the training set), and there were 35(60.3%) patients in the I-II stage and 23(39.7%) in III-IV stage. According to molecular typing, patients including 18(31%) cases of TNBC molecular subtype, 10(17.2%) cases of Luminal a type, 17(29.3%) cases of Luminal B type and 13(22.5%) cases of Her-2 positive. The validation set data is also displayed in the **Table 1**. And we compared the clinicopathological characteristics between the training and validation cohorts, all the variables were comparable (P>0.05). Complete table of baseline characteristics (**Supplementary Table 1**) and other laboratory test results (**Supplementary Table 2**) are listed in **Supplementary Data**.

**Table 1:** Characteristics of Breast Cancer patients in the training cohort and validation cohort.

Variables	Training cohort N (%)	Validation cohort N (%)	P value
Age			0.146
≤53	30 (51.7)	9 (34.6)	
>53	28 (48.3)	17 (65.4)	
Stage			0.125
I-II	35 (60.3)	11 (42.3)	
III-IV	23 (39.7)	15 (57.7)	
Molecular subtype			0.211
TNBC	18 (31)	7 (27)	
Luminal A	10 (17.2)	8 (30.7)	
Luminal B	17 (29.3)	3 (11.6)	
HER-2 positive	13 (22.5)	8 (30.7)	

### Development and validation of the cytokine-related signature

**Figure 1:**
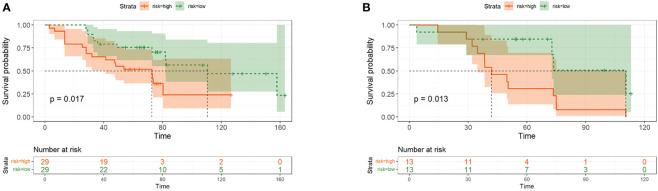
The Overall survival curves of the low-risk and high-risk groups in the Training Cohort **(A)** and Validation Cohort **(B)**.

**Figure 2:**
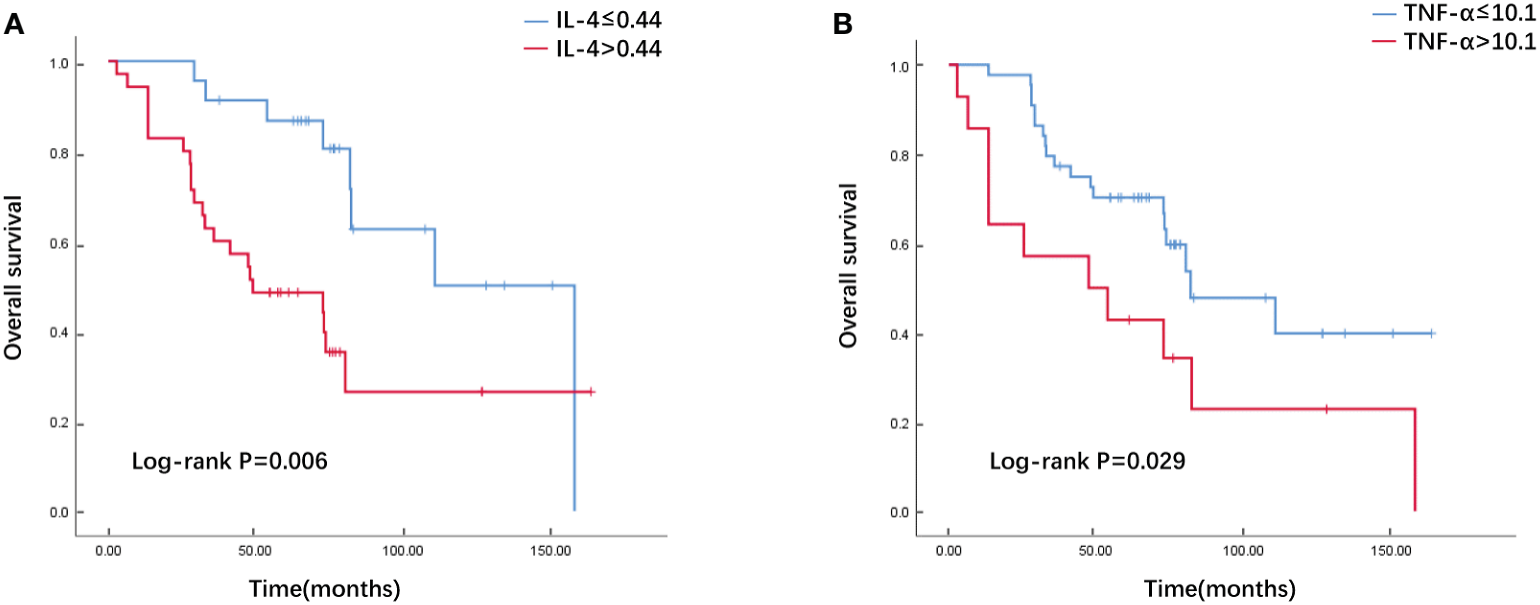
The Overall survival curves of breast cancer patients with high and low serum IL-4 **(A)**/TNF-α **(B)** levels in the training cohort.

Through multivariate Cox regression analysis and “step” function in R, a prediction model based on cytokine-related riskscore is obtained. The results showed that IL4 (HR=2.693, 95%CI 1.63-4.47, P<0.01) and TNF-α (HR=1.038, 95%CI 1.01-1.07, P<0.01) were powerful prognostic factors, and then we constructed the riskscore model. According to their coeficients and expression levels, we calculated riskscore for each patient in the training set as follows: riskscore =IL-4*0.99069 + TNF-α*0.03683. We used the median riskscore to divide the training set into risk-high group and risk-low group for survival analysis. The risk-high group had more deaths and shorter survival time than the risk-low group (**Figure 1A**). Significant differences in survival were detected between the two groups (P=0.017). The riskscore of the validation set was calculated according to the formula above. Next, the validation set is divided into high and low risk groups based on the median riskscore. The Kaplan–Meier analysis showed that patients in the low-risk group exhibited a significantly better OS than high-risk patients, indicating that the model had a good predictive eficacy (**Figure 1B**). We further analyzed the effect of serum IL-4 and TNF-α on OS and divided these two cytokines into high and low groups by calculating the cutoff value using Youden index. Our data revealed that both high serum IL-4 and TNF-α were indicative of poorer survival outcomes in comparison to low groups in the training set (P=0.006 and P=0.029, **Figure 2**).

### Survival analysis in subgroups

To evaluate the prognostic predictive value of the cytokine-related riskscore, a stratified subgroup analysis was performed according to clinical characteristics, as shown in **Supplementary Figure 1**. Both in young and old populations, the high-risk group had a poorer prognosis (**Supplementary Figure 1A**, age ≤ 53, P=0.017; age>53, P=0.047). And for patients with different tumor stages, the low-risk group had a better prognosis (**Supplementary Figure 1B**, stage I/II, P=0.067; stage III/IV, P=0.014), however, for patients in stage I/II, this difference was not significant. In different molecular types, the survival prognosis of high and low risk groups has some differences (**Supplementary Figure 1C**). In TNBC and HER2-positive patients, OS was significantly worse in the high-risk group than in the low-risk group (TNBC, P=0.011; HER2-positive, P=0.041). But for Luminal A and Luminal B patients, there was essentially no difference in OS between the two groups (Luminal A, P=0.726; Luminal B, P=0.49).

### Establishment and validation of a nomogram

**Figure 3:**
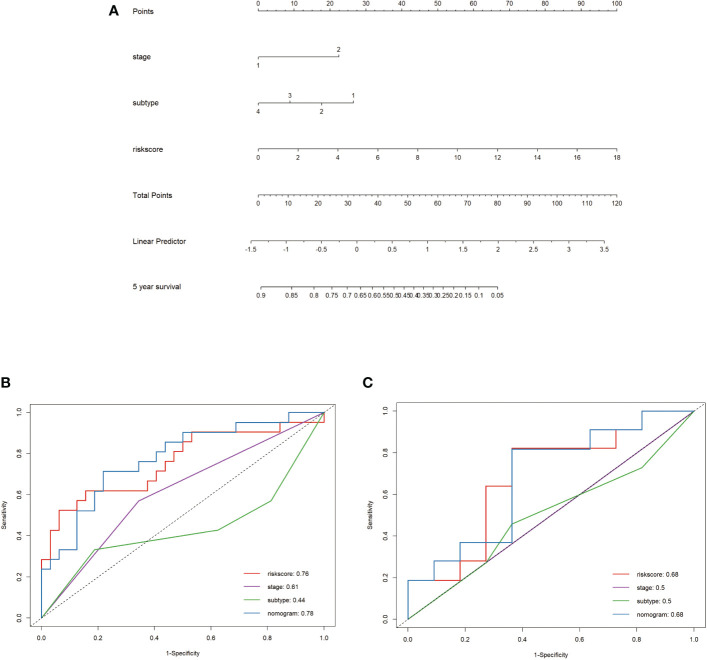
Nomogram for predicting 5-year OS in the training set **(A)**; ROC curves for 5-year OS based on the nomogram, riskscore, and other clinical traits in the Training Cohort **(B)** and Validation Cohort **(C)**.

Univariate and multivariate Cox regression analyses were used to assess whether cytokine-related riskscore could be used as an independent prognostic factor. According to the result of the univariate Cox hazard model, stage (P=0.001), Luminal B subtype (P=0.01) and riskscore (P<0.001) were significantly related to OS. After considering these factors, multivariate Cox regression analysis showed that cytokine-related riskscore was an independent predictor of poor survival in the training set (HR=1.2, P=0.003 **Table 2**). Then in the validation set, through the univariate Cox regression analysis, only cytokine-related riskscore was proven to be independent risk factors to predict overall survival (HR=1.6, P=0.023 **Table 3**). After adding all factors to multivariate Cox regression, riskscore (HR=2.25, P=0.031 **Table 3**) was still an independent prognostic factor. Subsequently, a nomogram of tumor stage, molecular subtype and riskscore was created to predict the 5-year OS of BC patients in the training cohort. (**Figure 3A**) The C-index of this nomogram was 0.79. The nomogram scores of the training and validation sets were then calculated using the “formula_rd” function in the “nomogramFormula” package. To compare the predictive value of the nomogram with the riskscore and other available clinical characteristics, ROC curves for 5-year OS were generated in the training set and validation set respectively. As shown in **Figure 3** the nomogram model had larger AUCs compared to other metrics. In the training set (**Figure 3B**), the 5-year AUCs for nomogram, riskscore, stage, and subtype were 0.78, 0.76, 0.61, and 0.44; in the validation set (**Figure 3C**), were 0.68, 0.68, 0.5, and 0.5.

**Table 2:** Univariate and multivariate cox analyses in the training cohort.

	Univariate		Multivariate	
Variables	HR (95% CI)	P-value	HR (95% CI)	P-value
Age	1.01 (0.97-1.05)	0.56		
Stage				
I-II				
III-IV	3.45 (1.62-7.34)	0.001*	2.67 (1.18-6.03)	0.019*
Molecular Subtype				
TNBC		0.07		0.12
Luminal A	0.52 (0.17-1.58)	0.25	0.53 (0.17-1.61)	0.26
Luminal B	0.25 (0.09-0.72)	0.01*	0.29 (0.1-0.82)	0.02*
HER-2 positive	0.75 (0.30-1.88)	0.54	0.58 (0.17-1.95)	0.381
Riskscore	1.24 (1.12-1.37)	<0.001*	1.2 (1.07-1.36)	0.003*

*P value <0.05 was considered significant.

**Table 3:** Univariate and Multivariate Cox Analyses in the Validation Cohort.

	Univariable		Multivariable	
Variables	HR (95% CI)	P-value	HR (95% CI)	P-value
Age	1 (0.94-1.06)	0.95	0.96 (0.9-1.03)	0.249
Stage				
I-II				
III-IV	1.81 (0.64-5.08)	0.26	2.12 (0.51-8.81)	0.3
Subtype				
TNBC		0.668		0.16
Luminal A	0.47 (0.13-1.71)	0.25	0.15 (0.02-0.95)	0.04*
Luminal B	0.42 (0.07-2.41)	0.328	0.11 (0.01-1.32)	0.08
HER-2 positive	0.67 (0.12-2.35)	0.526	0.28 (0.06-1.38)	0.118
Riskscore	1.6 (1.07-2.4)	0.023*	2.25 (1.08-4.69)	0.031*

*P value <0.05 was considered significant.

### Correlation analysis between cytokines and blood indicators

**Figure 4:**
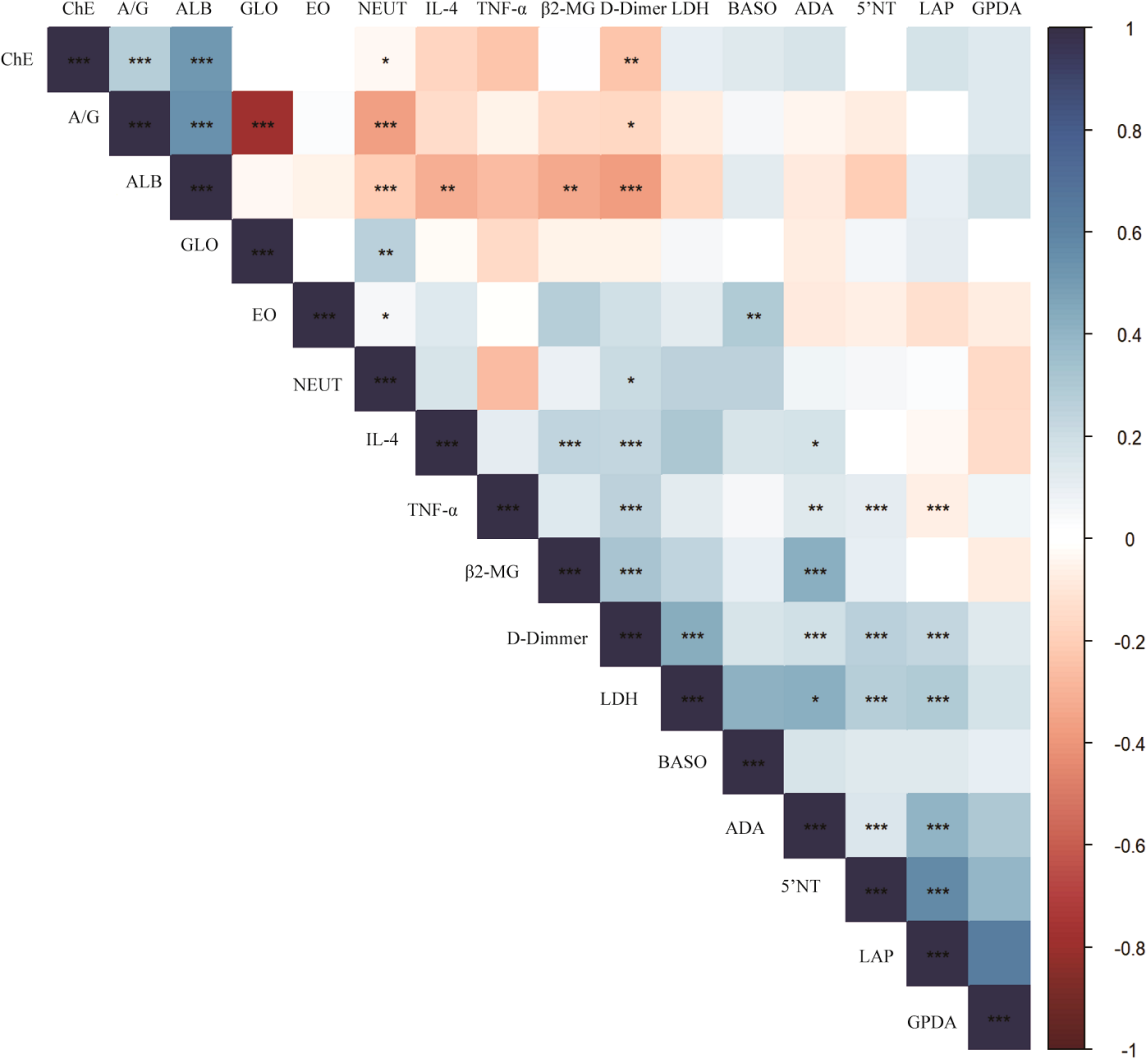
Correlation between cytokines and blood test results in breast cancer. P-value<0.05 is marked with *, P-value<0.01 is marked with **, P-value<0.001 is marked with ***.

As in the heatmap, the liver and kidney function, coagulation factors and routine blood test results of 84 breast cancer blood samples were retrospectively analyzed to explore the correlation between the two cytokines, IL-4 and TNF-α, the statistically significant results are marked with * (**Figure 4**). IL4 was negatively correlated with ALB (Spearman r = - 0.311, p <0.01) and positively correlated with β2-MG (Spearman r = 0.246, p <0.001), D-Dimer (Spearman r = 0.234, p <0.001) and ADA (Spearman r = 0.177, p <0.01). TNF-α was negatively correlated with LAP (Spearman r = - 0.067, p <0.001) and positively correlated with 5’NT (Spearman r = 0.118, p <0.001), D-Dimer (Spearman r = 0.259, p <0.001) and ADA (Spearman r = 0.14, p <0.01).

### Survival analysis based on Kaplan-Meier plotter database

**Figure 5:**
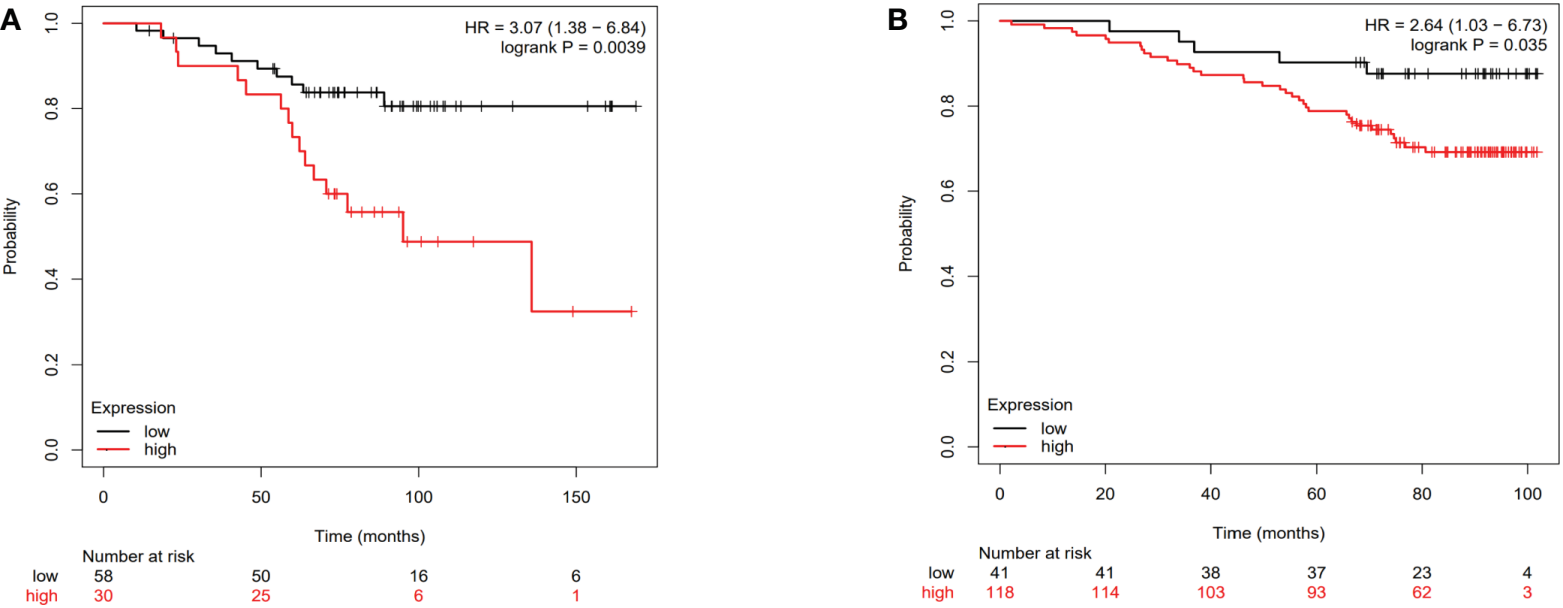
The Overall survival curves of breast cancer patients according to IL-4 **(A)** and TNFA **(B)** gene expression levels in the public dataset.

The Kaplan-Meier plotter public mRNA expression dataset was applied to analyze the relationship between IL-4 and TNFA mRNA expression levels and prognosis. The OS of patients with low IL-4 (**Figure 5A**, HR=3.07, P=0.039) and TNFA (**Figure 5B**, HR=2.64, P=0.035) gene expression were significantly better than in patients with high gene expression divided by the median value.

## Discussion

In this study, based on the detected levels of cytokines in serum, we screened out two cytokines by Cox analysis and “step” function to constructed a riskscore model related to breast cancer prognosis. In both training and validation cohorts, we found that the riskscore for IL-4 and TNF-α had a detrimental effect on overall survival in breast cancer. Immediately after, we established a nomogram containing tumor stage, molecular subtype and riskscore, and this nomogram has better prediction effect compared with cytokine-related riskscore.

IL-4 belongs to the interleukin family and interleukins are small molecular proteins secreted mainly by CD3 + and CD4 + T lymphocytes, which mediate the necessary interactions for the progress of cancer cells (14). Il-4 is called “prototype immunoregulatory cytokine”. Like many cytokines, it can affect a variety of target cells in various ways and plays an important role in regulating antibody production, hematopoiesis, inflammation and the development of efficacy T cells response (15). Cytokines can regulate key interactions between immune and non-immune cells in the tumor microenvironment (16).A study proved that IL-4 acts as a survival factor for cancer cells including breast cancer by increasing the expression of anti-apoptotic molecules, making them resistant to induction of apoptosis (17). A study in 1992 concluded that IL-4 is a non-autocrine inhibitor of breast cancer and can inhibit tumor proliferation (18). However, there are some discrepancies between the available studies on the correlation between serum cytokines and breast cancer prognosis. Our findings differ from these results. We obtained the cutoff value of IL-4 by calculating the Youden index and divided the patients into high and low expression groups. The Kaplan-Meier curve showed that patients in the high serum level group had a shorter survival time. The same results were verified in the Kaplan-Meier plotter database on mRNA level. As well as being a risk factor for breast cancer patients, DeNardo DG found that in murine breast cancer models, IL-4 produced by tumor infiltrating CD4 + T cells stimulate M1 to M2 transition of TAMs (Tumor-associated macrophages), thus supporting lung metastasis of breast cancer cells (19).

Tumor necrosis factor α belongs to tumor necrosis factor/tumor necrosis factor receptor (TNF/TNFR) superfamily cytokines and belongs to type II transmembrane protein (20). TNF-α is one of the most important pro-inflammatory cytokines in breast cancer TME, which is secreted by stromal cells (mainly M1 TAM) and cancer cells themselves. TNF-α plays a dual role in cancer and can enhance or inhibit tumor progression depending on the discovery of its specific cellular environment (21).High serum TNF-α remains an independent prognostic factor for poor overall survival in a case-control study of primary breast cancer patients (22). Which was similar to our study results. In our research, after adding age, stage and molecular type to the Cox analysis, the riskscore composed of IL-4 and TNF-α was still identified as a risk factor for OS in both the training and validation sets.

Cytokines have been reported to play a key role in the immune response to cancer cells. Several studies have been conducted to explore the correlation between serum cytokine levels and breast cancer immunity. At present, some studies have reported that cytokines can be used as a prognostic factor in breast cancer treatment. A study of the response to neoadjuvant chemotherapy in locally advanced breast cancer showed that serum TNF-α levels were significantly lower both in partial and complete response compared to pre-treatment (p < 0.01), there was also a significant difference in TNF-α levels between complete and partial response after treatment (23). In univariate and multivariate analyses, low TGF-β1 and TNF-α and high IFN-γ were associated with significantly lower risk ratios for disease progression, and serum TGF-β1, TNF-α, and IFN-γ levels may be potential prognostic biomarkers for breast cancer patients with bone metastases treated with taxane and zoledronic acid chemotherapy (24). In immunotherapy, TNF-α induced mucin 4 expression in HER2-positive breast cancer can cause resistance to trastuzumab (25). Increased IL4 levels found to be associated with better survival in small cell lung cancer patients receiving ipilimumab immunotherapy (26).

In clinical practice, we can combine cytokines and blood indicator analysis to find the cytokine that is related to the blood indicator, by detecting the expression level of this cytokine to prompt us to predict the outcome of patients. We have collected several blood tests results from patients record and correlated them with the two cytokines. In our study, the results showed that ALB was negatively correlated with IL-4, while β2-MG, D-Dimer and ADA were significantly positively correlated with IL-4. As for TNF-α, it was positively associated with D-Dimmer, ADA and 5’ NT and negatively associated with LAP. Albumin is produced by the liver and has been used to assess severity of disease, disease progression and prognosis. Christopher G Lis et al. found that albumin levels<3.5g/dL had a threefold increased risk for death, and baseline serum albumin levels were a powerful prognostic variable (27).A study showed that serum albumin, lactate dehydrogenase and total bilirubin were significantly associated with 5-year OS in multivariate Cox analysis in breast cancer (11). However, there are few studies on the prognostic value of β2-mg, D-dimmer, 5’ NT, ADA and LAP in breast cancer at present. D-dimer is a biomarker used to indicate clotting and fibrinolytic activity and is more widely used in predicting the risk of thrombosis. There were two studies found significant reductions in serum 5’-NT levels after mastectomy, and the 1995 study also found decreases in serum ADA levels (28, 29). Another research suggested that ADA activity in breast tissue can be an indicator for the differential diagnosis of benign and malignant breast disease. Due to the age of these studies and the lack of more verification, the conclusions need to be more supported in future research. In the next study, we will further explore the effect of cytokines combined with blood markers on the prognosis of breast cancer.

For the nomogram we build, age was not included, because it has not been verified as an independent prognostic factor for OS in BC patients in multivariate Cox analysis. However, in the subgroup analyses we could see that the prognosis of patients with high-risk was poorer and significantly different in both young and old populations, suggesting that there is also a correlation between age and the riskscore. In the subgroup analysis, we can observe that for patients with tumor stage III/IV, the low-risk group has a better prognosis. And among the different molecular subtypes, TNBC and HER2-positive patients with high-risk had a shorter survival. This suggests that cytokine-related riskscore correlates with patient age, tumor stage and molecular subtype, but it has less predictive value for patients with early stage tumors and Luminal type.

One limitation of our study is that we did not include healthy controls and lacked data to compare the differences in cytokine levels between patients and normal individuals. Several studies have found that there were statistical differences in cytokine levels between cancer patients and healthy controls. Significantly elevated levels of IL-4, IL-8 and IL-9 were observed in colorectal cancer (30, 31). And IL-4 was also found higher in breast cancer patients (32). Ultimately, due to the small sample size, our findings need to be validated in a larger cohort and multicenter study.

## Conclusion

Taken together, our study explored the role of cytokine-related genes in the prognosis of BC patients. We analyzed and validated a cytokine-related riskscore, and developed a prognostic nomogram combining tumor stage, molecular subtype and riskscore for clinical OS prediction. Then analyzed the correlation between cytokines and blood test indicators. It is hoped that this nomogram will provide individualized prediction of survival for BC patients.

## Data availability statement

The original contributions presented in the study are included in the article/**Supplementary Material**. Further inquiries can be directed to the corresponding authors.

## Ethics statement

The studies involving human participants were reviewed and approved by the Ethical Committee of the Tianjin Medical University Cancer Institute and Hospital. The patients/participants provided their written informed consent to participate in this study.

## Author contributions

YZ: Conceptualization, data collection, formal analysis, and methodology and writing. YZ and YH: data analysis and revised the manuscript. YH and CC: Data curation and experiments. JZ, XY, and YL: Collection and assembly of data. WZ and Y-ZC: Conceptualization and manuscript review and editing. All authors contributed to the article and approved the submitted version.
