# Supplementary material for: Development of a nomogram based on serum cytokine-related riskscore in breast cancer
Source: Front Oncol. 2023 Mar 7;13:1146463. doi: 10.3389/fonc.2023.1146463 (PMC10062183; doi:10.3389/fonc.2023.1146463)
Supplement: Supplementary file 1 [file DataSheet_1.docx]

Supplementary Material

Development of a nomogram based on serum cytokine-related riskscore in Breast Cancer

**Ye Zhu, Yang He, Chong Chen, Jingyi Zhang, Xin Yang, Yuqing Lu, Yong-Zi Chen*，Weipeng Zhao***

***Correspondence:**

Weipeng Zhao

zhaoweipeng@tjmuch.com

Yong-Zi Chen

yzchen@tmu.edu.cn

| **Supplementary Table 1.** The Baseline Clinicopathological Characteristics | |
| --- | --- |
| **Variables** | **Value** |
| **Age (years)** | 55 (45.25-59) |
| **Tumor Stage** |  |
| Ⅰ-Ⅱ | 46 (54.8%) |
| Ⅲ-Ⅳ | 38 (45.2%) |
| **T stage** |  |
| T1 | 30 (35.7%) |
| T2 | 42 (50%) |
| T3 | 4 (4.8%) |
| T4 | 8 (9.5%) |
| **N stage** |  |
| N0 | 30 (35.7%) |
| N1 | 23 (27.4%) |
| N2 | 8 (9.5%) |
| N3 | 23 (27.4%) |
| **M stage** |  |
| M0 | 68 (81%) |
| M1 | 16 (19%) |
| **Tumor subtype** |  |
| TNBC | 25 (29.8%) |
| Luminal A | 18 (21.4%) |
| Luminal B | 20 (23.8%) |
| HER-2 positive | 21 (25%) |
| **Ki67 expression level** |  |
| Low | 28 (33.3%) |
| High | 56 (66.7%) |
| Age was expressed as median (interquartile range) and others were expressed as percentage. | |
| T stage: T1: Tumor size≤2 cm; T2: 2 cm<Tumor size≤5 cm; T3: Tumor size>5 cm; T4: Any tumor size directly invades the chest wall or skin (excluding only dermal infiltration ). | |
| N stage (No. of lymph node metastases): N0: 0; N1: 1~3; N2: 4~9; N3: ≥10 | |
| M stage: M0: No distant metastasis; M1: Occurrence of distant metastases | |
| Ki67 expression levels: Low: < 20%; high: ≥ 20% | |

| **Supplementary Table 2.** The Laboratory Tests Results | |
| --- | --- |
| **Variables** | **Value** |
| **IL-1β (pg/ml)** | 1.02 (0.68-1.58) |
| **IL-2 (pg/ml)** | 0.6 (0.41-0.88) |
| **IL-4 (pg/ml)** | 0.73 (0.33-1.26) |
| **IL-5 (pg/ml)** | 0.32 (0.17-0.56) |
| **IL-6 (pg/ml)** | 6.15 (2.37-19.71) |
| **IL-8 (pg/ml)** | 49.47 (12.6-223.66) |
| **IL-10 (pg/ml)** | 2.2 (1.77-3.01) |
| **IL-12P70 (pg/ml)** | 1.59 (1.03-2.45) |
| **IL-17A (pg/ml)** | 4.28 (0.07-11.79) |
| **TNF-α (pg/ml)** | 3.51 (1.83-10.12) |
| **IFN-α (pg/ml)** | 0.95 (0.66-1.4) |
| **IFN-γ (pg/ml)** | 1.21 (0.75-2.28) |
| **NEUT (10^9^/L)** | 3.7 (2.41-4.44) |
| **EO (10^9^/L)** | 0.07 (0.02-0.1) |
| **BASO (10^9^/L)** | 0.02 (0.01-0.03) |
| **ALB (g/L)** | 43.45 (41.03-45.5) |
| **GLO (g/L)** | 31.5 (29.8-34.4) |
| **A/G (pg/ml)** | 1.35±0.23 |
| **ChE (U/L)** | 8180.8±1778.79 |
| **ADA (U/L)** | 5 (4-5) |
| **LDH (U/L)** | 253.5 (192-329.5) |
| **GPDA (U/L)** | 95 (69-95) |
| **5'NT (U/L)** | 11 (9-13.4) |
| **LAP (U/L)** | 58.5 (49-67) |
| **β2-MG (mg/L)** | 1.85±0.58 |
| **D-Dimer (ng/ml)** | 873.7 (430.64-961.88) |
| Normal data and non-normal data were expressed as mean ± standard deviation and median (interquartile range), respectively. | |

**
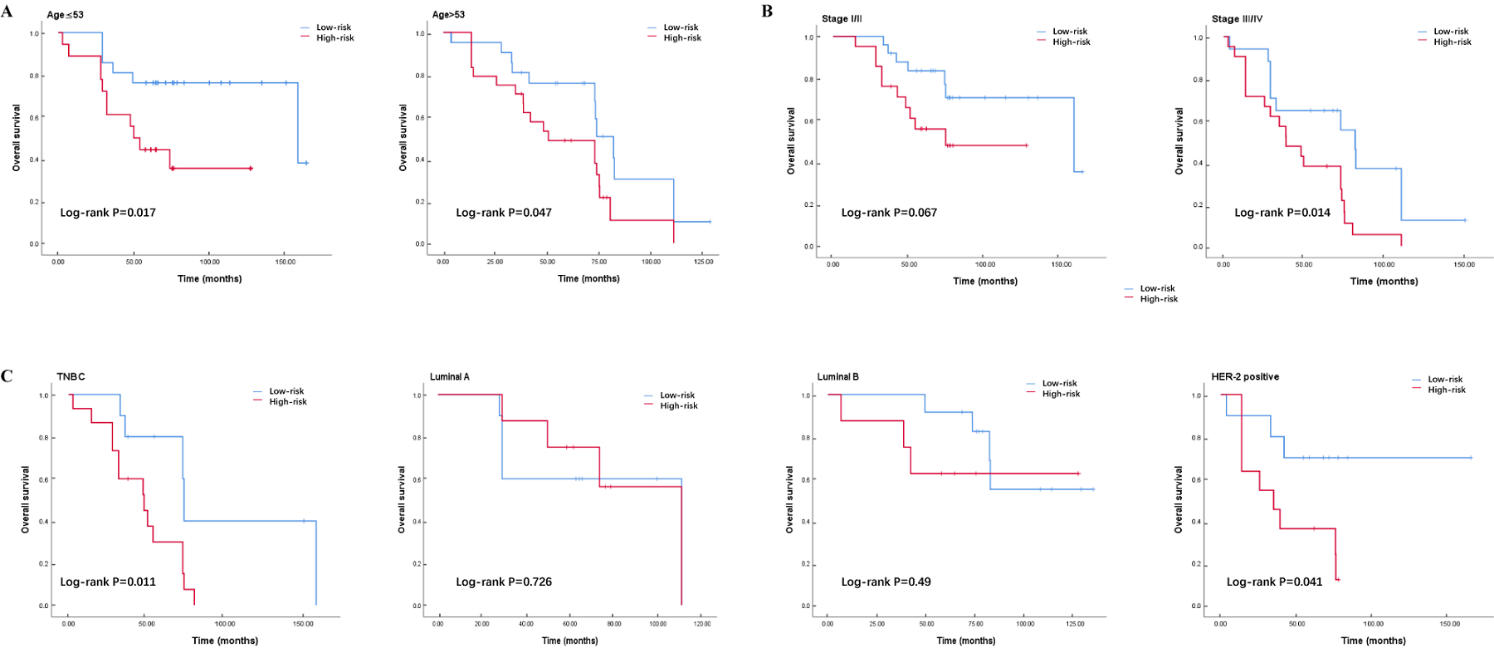
**

**Supplementary Figure 1.** Subgroup analyses of the cytokine-related riskscore according to age (A), tumor stage (B) and molecular subtype (C)
